# Th1/Th17 T cell Tissue-Resident Immunity Increases Protection, But Is Not Required in a Vaccine Strategy Against Genital Infection With *Chlamydia trachomatis*

**DOI:** 10.3389/fimmu.2021.790463

**Published:** 2021-12-02

**Authors:** Nina Dieu Nhien Tran Nguyen, Safia Guleed, Anja Weinreich Olsen, Frank Follmann, Jan Pravsgaard Christensen, Jes Dietrich

**Affiliations:** ^1^ Department of Infectious Disease Immunology, Statens Serum Institut, Copenhagen, Denmark; ^2^ Department of Immunology & Microbiology, University of Copenhagen, Copenhagen, Denmark

**Keywords:** vaccine, Th1, Th17, infection, Chlamydia

## Abstract

The requirement for vaccine-induced tissue-resident immunity for protection against one or repeated infections with *Chlamydia trachomatis (C.t.)* is still not fully resolved. In this study, our aim was to investigate to which degree tissue-resident Th1/Th17 T cells in the genital tract (GT) could add to the protection mediated by circulating immunity. Out of several mucosal vaccine strategies, a strategy termed SIM (for simultaneous intrauterine and parenteral immunization with CAF01 adjuvanted CTH522), was superior in generating genital tract tissue-resident Th1/Th17 T cell immunity. This led to a faster and stronger local CD4 T cell response post infection, consisting of multifunctional IFNγ/TNFα-producing Th1 T cells and IFNγ/TNFα/IL-17-producing Th17 T cells, and a faster recruitment of innate immune cells. Post infection, SIM animals showed an additional significant reduction in bacterial levels compared to mice having received only a parenteral vaccine. Nevertheless, the parenteral strategy reduced bacterial levels by 75%, and interestingly, post infection, these mice generated their own vaccine-derived genital tract tissue-resident memory Th1/Th17 T cells, which upon a subsequent infection showed as fast an activation in the genital tract, as observed in SIM mice. Furthermore, in contrast to after the first infection, both groups of mice now showed a similar infection-induced boost in local vaginal IgA and IgG titers. Thus, vaccine-induced resident immunity, generated pre-infection, led to an advantage in the response against the first infection, but not the second infection, suggesting that a parenteral vaccine strategy is a suitable vaccine strategy against infections with *Chlamydia trachomatis*.

## Introduction

*Chlamydia trachomatis (C.t.)* is the most common sexually transmitted bacterium with an estimated 127 million new cases occurring every year globally (1). *C.t.* is an obligate intracellular bacterium infecting both men and women. Genital infections are frequently asymptomatic and consequently left untreated. Untreated women can experience serious sequelae such as pelvic inflammatory disease that can lead to fatal ectopic pregnancy and infertility (2–4). Moreover, as repeated infections with *C.t.* are not uncommon (5), it has become clear that the goal of a vaccine against *Chlamydia* is to prevent not only a first infection, but also subsequent infections. To develop such a vaccine, a better understanding of the most optimal protective immune response is required.

Protective CD4 tissue-resident memory T (Trm) cells have been observed at barrier sites such as the lung and skin (6) and genital tract (GT) (7, 8). Trm cells rapidly respond to local antigen recognition by a cytokine release, which in turn facilitate recruitment of circulating memory T cells and innate cells (9–13). In the genital tract, Trm cells have been studied in both HSV (7) and *C.t.* models (8). Intravaginal immunisation has shown that CD4 T cells in the GT associated with macrophages to form clusters that elicited increased protection compared with circulating immunity (7). Topical application of CXCL9 and CXCL10 induced recruitment of vaccine-induced CD8 T cells into the GT, which showed superior protection compared to systemic vaccine-induced CD8 T cells recruited upon genital HSV challenge (14). Using a *C.t.* transcervical (TC) infection model, CD4 Trm cells showed superior protection compared to systemic CD4 T cells (8). These results show that Trm cells have an important protective potential, but they do not show if Trm cells are absolutely required in a vaccine strategy against *Chlamydia*. Moreover, the requirement for vaccine-induced Trm cells for protection against repeated infections has not been investigated. We recently showed that a parenteral vaccine inducing only systemic immunity, given prior to the first infection, induced protective systemic Th1/Th17 T cell immunity that could be measured between day 3 and 7 post infection (15). The question that we therefore addressed in the present study, was if and how the induction of vaccine specific tissue-resident Th1/Th17 Trm cells in the GT, prior to infection, could add to this response, and if pre-infection generated Trm cells would also add to the response against repeated infections. We used the *Chlamydia* vaccine antigen CTH522, formulated in the adjuvant CAF01, which possesses a unique property of inducing both Th1 and Th17 T cells (16–18).

## Materials And Methods

### Ethics Statement

Experiments were conducted in accordance with the regulations set forward by the Danish Ministry of Justice and animal protection committees by Danish Animal Experiments Inspectorate Permit 2020-15-0201-00637 and in compliance with European Community Directive 2010/63/EU of the European parliament and of the council of 22 September 2010 on the protection of animals used for scientific purposes, as well as Directive 86/609 and the U.S. Association for Laboratory Animal Care recommendations for the care and use of laboratory animals. The experiments were approved by a local animal protection committee at Statens Serum Institut, IACUC, headed by DVM Kristin Engelhart Illigen.

### Animals

Studies were performed with 6- to 8-week-old female B6C3F1 hybrid mice from Envigo, Scandinavia. Animals were housed in appropriate animal facilities at Statens Serum Institut and handled by authorized personnel.

### Bacteria Preparations and Transcervical Infection

*C.t.* SvD (ATCC) were grown in HeLa cells (ATCC) in RPMI 1640 media (Invitrogen) supplemented with 1%HEPES, 1% of Non-essential amino acids (NEAA) (MP Biomedicals), 1% L-Glutamin (Gibco) and 1% pyruvate (Gibco). The infected HeLa cells were grown for 2-3 days at 37°C at 5% CO_2_. Infected HeLa cells were harvested and *C.t*. were purified from the cells (19). Purified *C.t.* were resuspended in SPG buffer (250 mM Sucrose, 10 mM Na_2_HPO_4_, 5 mM L-glutamic acid) and divided into aliquots at a concentration of 2.7x10^7^ IFUs/ml. Aliquots were stored at -80°C

All mice were treated 10 and 3 days before infection with 50 mg of medroxyprogesteron to synchronize the murine estrous cycle. Mice were transcervically infected using a thin, exible probe: nonsurgical embryo transfer (NSET) device (Paratechs) to bypass the cervix and to inject bacteria directly into the uterine horn lumen.

### Antigens, Adjuvant and Immunization

Mice were immunized three times at two-week intervals with MOMP-based recombinant antigen CTH522 (17) (5µg per dose) formulated in CAF01 (DDA/TDB 250µg/50µg per dose). Subcutaneous administration was done by injecting 200µl of the vaccine at the base of the tail. Intrauterine administration was done in the same manner as TC inoculation with *C.t.* where 20 µl of the vaccine [5ug CTH522 in CAF01 (DDA/TDB 250µg/50µg per dose)] was administered in the uterine horn lumen by using a NSET device. Non vaccinated mice received no treatment.

### Bacterial Burden

To quantify the bacterial burden in the infected mice, the upper genital tract was swabbed (one stick for each uterine horn). Swabsticks were stored at -80°C in 600 mL SPG buffer (250 mM Sucrose, 10 mM Na2HPO4, 5 mM L-glutamic acid) with plastic beads. For cell passage, 80,000 McCoy cells (ATCC) in infection media (RPMI 1640 (Invitrogen), 1 %HEPES (Gibco), 5 % FBS (VWR), 0.01% Gentamicin (Gibco), 0.5 % Glucose) were seeded in a 48-well plate (Costar) and incubated at 37°C with 5 % CO^2^ overnight. When the samples reached 85-90% confluence cell media was aspirated and 0.2 ml infection medium was added to the wells and incubated at 37°C with 5 % CO^2^. Undiluted and 1:2 diluted samples were added to the wells and centrifuged at 700 G for 1 hour with no brake at room temperature. Afterwards, the plates where placed at 37°C with 5% CO^2^ for 2 hours. Next, supernatants were aspirated and 0.5 ml infection medium with 1:1000 Cycloheximide (Sigma) were added to the wells and incubated for one day at 37°C. The cells were then fixated with 0.4 mL 96 % ethanol per well and kept in 0.4 mL 1xPBS overnight at 4°C. The nucleius were dyed with 0.2 mL/well propidiumiodid (Sigma) (solution 1:2) and 0.25 mL/well of sterile-filtrated diluted rabbit anti-SerovarD MOMP antibody (in house) was added to label the inclusion bodies and incubated for 1 hour at room temperature. Next the cells were incubated at room temperature for 1 hour with Alexa Flour 488 conjugated secondary antibody goat anti-rabbit IgG (0.1mL/well, Life Technologies) diluted 1:500 in 1xPBS 1 % BSA. IFUs were quantified using ImageExpress® PICO (Molecular Devices) and the CellReporterXpress® software.

### Total IgG, IgG Subclasses and IgA-ELISA

Nunc MaxiSorp 96-well plates (Sigma-Aldrich) were coated with CTH522 antigen (1µg/ml) diluted in carbonate buffer overnight at 4°C. For detection of IgG antibodies the plates were blocked with 1xPBS (made from 10x PBS, Gibco Invitrogen) with 2% BSA for 2 hours. For detection of IgA antibodies the plates were blocked with 1% skim milk and 0.05% Tween (Merck). The plates were afterwards washed 3 times with washing buffer (PBS+0.2% Tween). The samples were titrated with 1% BSA as indicated in each figure, and incubated for 2 hours at room temperature. The samples were then incubated for 1 hour at room temperature with secondary HRP-conjugated against IgG antibodies: rabbit anti-mouse IgG (H+L) (AH Diagnostics), goat anti-mouse IgG1 (Southern Biotech), rabbit anti-mouse IgG2a (AH Diagnostics), rabbit anti-mouse IgG2b (AH Diagnostics) or goat anti-mouse IgG2c (Southern Biotech). For IgA detection the samples were incubated with goat anti-mouse IgA-biotin for 1 hour at room temperature followed by incubation with Streptavidin-HRP antibody for 30 minutes at room temperature. The samples were developed by adding 3, 3’, 5, 5’-tetramethylbenzidine (TMB PLUS2^®^, Kementec). After 5-15 minutes the reactions were stopped by adding 0.5 M H_2_SO_4_ sulfuric acid (Honeywell Fluka™). Plates were read at 450 nm and with a background correction at 620 nm by using SunriseTM Absorbance Reader (Tecan Life Sciences).

For vaginal IgG/IgA, vaginal swabs were collected for IgA/total IgG quantification. Swabsticks were stored at -80°C in 600 ml SPG buffer (250 mM Sucrose, 10 mM Na2HPO4, 5 mM L-glutamic acid) with plastic beads. Mice are not exsanguinated before collecting vaginal swabs.

### Sandwich IFNγ and IL-17A ELISA

Single-cell suspension were adjusted to 10^5^ cells per well in 96 well round (U) Bottom plate (ThermoFisher). The cells were stimulated with 5 µg/ml antigen or peptides and incubated at 37°C at 5% CO_2_ for stimulation. Supernatants were harvested after 72 hours of stimulation. Each sample was done in triplicates. Nunc MaxiSorp 96-well plates (Sigma-Aldrich) were coated with rat anti-mouse IFNg (1:1000, BD Pharmingen, clone R46A2) or rat anti-mouse IL-17 (1:500, Biolegend, clone TC11-18H10.1) in carbonate buffer (SSI Diagnostica) and incubated overnight at 4°C. The plates were blocked with 1xPBS with 2% skim milk powder (Natur Drogeriet) for 2 hours at room temperature. Supernatants and recombinant IFNγ/IL-17A (Biolegend) were diluted in PBS 2% Bovine Serum Albumin (BSA) (Sigma-Aldrich), and plates were incubated for 2 hours at room temperature. The samples were incubated with biotin rat anti-mouse IFNγ (1:5000, BD Pharmingen, clone XMG1.2) or biotin anti-mouse IL-17A (1:2000, Biolegend, clone TC11-8H4) in 1xPBS 1% BSA for 1 hour at room temperature. Plates were then incubated for 30 minutes in 1:5000 diluted streptavidin-conjugated horseradish peroxidase (HRP) (BD Pharmingen) in 1xPBS 1% BSA. The samples were developed by adding TMB Ready-to-use substrate (Kem-En-Tec Diagnostics) and the enzyme reaction was stopped after 5-15 minutes by using 0.5 M H_2_SO_4_. Plates were read at 450 nm and with a background correction at 620 nm by using SunriseTM Absorbance Reader (Tecan Life Sciences). Standard curves were generated using known concentrations of recombinant IFNγ and IL-17.

### Sample Collection and Cell Preparation

Samples were obtained from 4-12 mice per group (individually or pooled in groups of 2) in RPMI 1640 (Gibco Invitrogen). 3 minutes before euthanasia, 250 µl of anti-CD45.2 – fluorescein isothiocyanate (BD Pharmingen, clone 104, 1:100 dil.) were intravenously injected into the tail of the mice to label vascular leukocytes. Single-cell suspensions were created by homogenizing organs through a 100 µm nylon filter (Falcon). In addition. GTs were incubated before homogenization for 1 hour at 37°C CO_2_ in type IV collagenase (0.8 mg/ml) (Sigma), 30 minutes in DNAse I (Roche) (0.08 mg/ml). Before and after incubation GTs were processed with gentleMACS™ Dissociator (Miltenyi Biotec). Cell suspensions were centrifuged (700 x g, 5 min) and washed twice in RPMI 1640. For PBMC isolation from blood, the blood was kept in tubes with EDTA and diluted 1:1 in 1xPBS. The blood was added on top of Lympholyte^®^ solution and centrifuged at 800 x g for 20 minutes at room temperature. The PBMC layer was harvested and washed twice in RPMI 1641. Cell pellets from all organs were resuspended in RPMI-1640 (Gibco Invitrogen) supplemented with 5 × 10−5 M 2-mercaptoethanol, 1 mM glutamine, 1% pyruvate, 1% penicillin-streptomycin, 1% HEPES, and 10% FCS (Gibco Invitrogen).

### Flow Cytometry and Biodistribution Analysis

For intracellular cytokine staining, cells were stimulated for 1 hour in the presence of CTH522 antigen and 1 µg/ml of costimulatory antibodies CD28 (BD Pharmingen,clone: 37.51) and CD49d (BD Pharmingen, clone: 9C10 (MFR4.B)). Brefeldin A was added afterwards at a concentration of 200µg/ml to each sample and were subsequently incubated at 37°C for 5 hours and kept at 4°C until staining. Cell suspensions were Fc-blocked with anti-CD16/CD32 antibody (BD Pharmingen, clone 2.4G2, 1:100 dil.) for 10 min. at 4°C. Cells were stained with combinations of the following anti-mouse antibodies conjugated to fluorochromes (company, clone, dilution): α-CD4-BV786 (BD Horizon, GK 1.5, 1:600), α-CD44-Alexa fluor 700 (Biolegend, IM7, 1:150), α-CD8-BV421 (Biolegend, 53-6.7, 1:200), α-CD69-PE-Cy7 (BD Pharmingen, H1.2F3, 1:200), α-IL-2-APC-Cy7 (BD Pharmingen, JES6-5H4, 1:200), α-IFNγ-PE (BD Pharmingen, XMG1.2, 1:200), α-TNF-APC (BD Pharmingen, MP6-XT22, 1:200), α-IL-17-PerCP-Cy5.5 (Invitrogen, eBIO17B7, 45-7177-82, 1:200), α-CD11b-APC-Cy7 (Biolegend, M1/70, 1:100), α-CD11c-APC (BD Pharmingen, HL3, 1:100), α-Ly6G-PE (BD Pharmingen, HL3, 1:100), and Viability-eFluor506 (Invitrogen, 1:500).

The stained cells were analyzed using a Flow cytometer (BD LSRFortessa, BD Bioscience) and FlowJo Software (version 10). To analyse the cells in the organs we excluded doublets on forward scatter height (FSC-H) and FSC area (FSC-A) plot, excluded cell debris on side scatter height (SSC-H) and FSC-H and last excluded “dead” cells using the viability marker. Leukocytes were divided into CD4 T cells (CD4^+^), CD8 T cells (CD8^+^), neutrophils (Ly6G^+^ CD11b^+^), macrophages (Ly6G^-^CD11b^+^CD11c^-^) and dendritic cells (Ly6G^-^CD11b^+^CD11c^+^MHC-II^+^).

### Statistical Analysis

Cell percentages and IFU counts among groups were analyzed by a one-way ANOVA followed by Tukey’s multiple comparison test, if more than two groups were compared as indicated in the figure legend. If only two groups were compared an unpaired student t test was used to determine significance. Prism version 8 software (GraphPad) was used for analysis. A p value of ≤0.05 was considered a significant difference.

## Results

### Only a Mucosal SIM Vaccination Strategy Induces Local Genital Tract T Cells and IgA Pre-Challenge

We first compared three vaccine strategies, a parenteral (subcutaneous, SC) CAF01 adjuvanted administration, an intrauterine mucosal CAF01 adjuvanted administration (IU) and a combination of the two given simultaneously (SIM). The latter strategy was chosen as we previously showed that combining a local immunization with a parenteral immunization led to a strong local T cell response (20). For the vaccine antigen we chose CTH522, which has previously been shown to protect against a vaginal or TC infection with *C.t* (15, 17 *) * and to generate both Th1 and Th17 T cells (16, 18).

**Figure 1:**
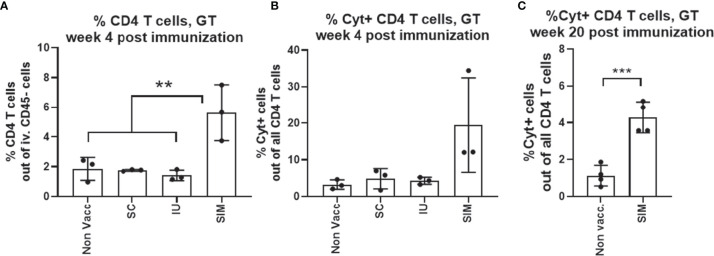
Only mucosal SIM vaccine strategy induces local T cells pre-challenge. Groups of female B6C3F1 mice (n=6-8, pooled pairwise) were vaccinated three times as indicated with two weeks intervals. The antigen was CTH522 and the administration route is indicated in the figure. **(A)** Percentage of CD4 T cells among all cells in the GT were determined by flow cytometry. **(B)** Among the CD4+ T cells, percentages of cytokine (IFNγ, IL-2, IL-17, and/or TNFα) positive antigen-specific CD44+ CD4+ T cells were determined. SIM Cyt+ indicate only the cytokine positive cells in the genital tract of SIM vaccinated mice. **(C)** Percentages of cytokine (IFNγ, IL-2, IL-17, and/or TNFα) positive antigen-specific CD44+ CD4+ T cells at week 20 post last vaccination. Bars indicate means ± SD. Statistical significance was evaluated by an ANOVA test followed by Tukey’s multiple comparisons. *p < 0.05 or by an unpaired t test **(C)**. **p < 0.001, ***p < 0.0001. The data shown are representative of 3 experiments **(A, B)** or 1 experiment **(C)**.

Female B6C3F1 mice were vaccinated three times, at two weeks intervals with CTH522/CAF01, and analyzed at week 6 post last vaccination. To measure leukocytes in the genital tract tissue, and not intravascular leukocytes, mice were subjected to *in vivo* intravascular staining by injecting fluorescein isothiocyanate (FITC)-labeled anti-CD45 monoclonal antibody (mAb) intravenously (iv.CD45) 3 min before the mice were euthanized. This selectively stains intravascular, but not tissue leukocytes [as described by Anderson et al. (21)] and enabled us to exclude intravascular cells from the subsequent flow cytometry analysis. Gating strategy is shown in **Supplementary Figure 1**. We first analyzed the percentage of CD4 positive T cells out of all iv.CD45 negative cells in the upper genital tract (uGT). 5.6% of all cells in the GT were CD4 T cells in the SIM group, and out of these CD4 T cells, 19.5% expressed either IFNγ, TNFα, IL2 or IL-17 (**Figures 1A, B**). Of the cytokine positive (Cyt+) CD44+CD4+ T cells, more than 80% also expressed CD69, a marker for resident T cells (data not shown). Even 20 weeks after the last vaccination, we could measure a population of cytokine positive CD4 T cells in the GT (**Figure 1C**). In the SC and IU group we did not detect an increase in neither the percentage of CD4 T cells, nor the percentage of Cyt+ CD4 T cells in the GT, compared to non vaccinated mice (**Figures 1A, B**).

**Figure 2:**
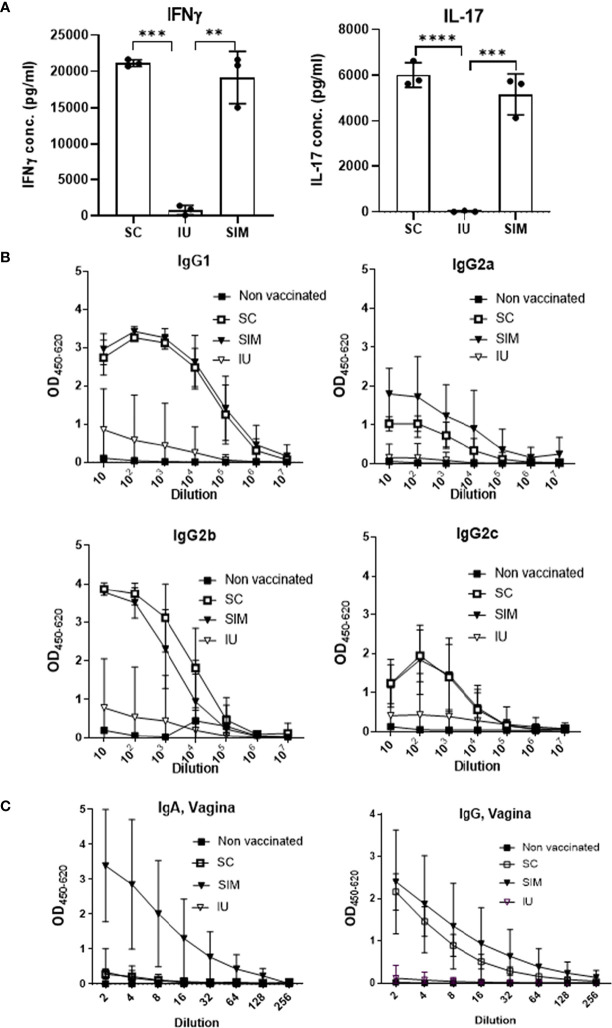
Systemic and local response pre-infection. **(A)** Groups of female B6C3F1 mice (n=6, pooled pairwise) were vaccinated three times as indicated with two weeks intervals. 6 weeks post last vaccination splenocytes were stimulated with CTH522 for 72 hours and secreted IFNγ or IL-17 in supernatant was measured. **(B)** IgG1/IgG2a/IgG2b/IgG2c levels were determined in the serum. **(C)** Total IgG and IgA levels in the vagina. Points and bars indicate means ± SD. Statistical significance was evaluated by an ANOVA test followed by Tukey’s multiple comparisons. **p < 0.01. ***p < 0.001, ****p < 0.0001. **(A, B)** Are representative of 3 experiments, and **(C)** of two experiments.

To compare the systemic T cell response, splenocytes were stimulated *in vitro* with CTH522 and secretion of IFNγ and IL-17 was measured. The results showed that stimulated cells from SC or SIM vaccinated animals released both IFNγ and IL-17. In contrast, mice from the IU group did not show a systemic CMI response (**Figure 2A**). We also tested the serum IgG. In contrast to the IU group, both the SC and SIM group showed a high CTH522 specific IgG titer in serum, consisting of both IgG1 and IgG2 subclasses, in agreement with the subclass distribution normally associated with the CAF01 adjuvant (**Figure 2B**). Finally, we also tested vaginal fluids for IgA and IgG. SIM mice showed the highest IgA response, whereas SC and IU mice showed no IgA at this time point. Both SC and SIM animals did, however contain vaginal IgG (**Figure 2C**).

Thus, only the CAF01 adjuvanted SIM vaccine strategy was able to induce both an increased local Th1/Th17 T cell response in the genital tract, combined with systemic IgG and local IgG and IgA.

### The Th1/Th17 Immune Response in the Genital Tract Post Infection

**Figure 3:**
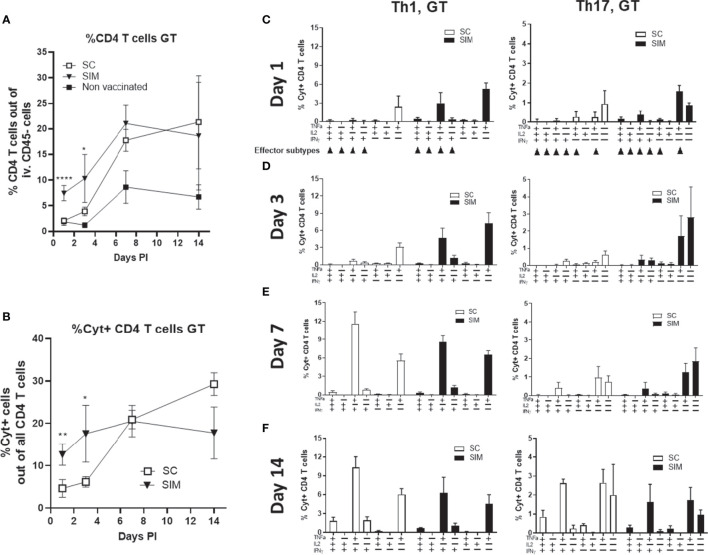
Day 1-14 post infection Th1 and Th17 cytokine subsets. Female B6C3F1 mice (n=8, pooled pairwise) were vaccinated three times as indicated with two weeks intervals. 6 weeks post immunization the mice received a TC infection with 1.5x10^3^ IFU of *C.t.* SvD. **(A, B)** The percentages of CD4 T cells, and cytokine positive (Cyt+) (IFNγ, IL-2, IL-17, and/or TNFα) in uGT at day 1-14 post infection were analyzed by flow cytometry. **(C–F)** Percentages of IL-17 negative CD4 T cells (Th1) and IL-17 positive CD4 cells (Th17) out of all CD4+ T cells in the uGT were analyzed by flow cytometry for the frequency of cytokine subsets (expression of TNFα, IL-2, IFNγ and/or IL-17). Points and bars indicate means ± SD. Small arrows in **(C)** indicate Th1 or Th17 effector subsets. In A and B statistical significance between SC and SIM animals was evaluated by an ANOVA test followed by Tukey’s multiple comparisons using Graphpad Prism 8.3.0. *p < 0.05. **p < 0.01, ****p < 0.0001 **(A)** or by a t-test **(B)**. The data shown are representative of 2-3 experiments.

We next examined how the vaccine-induced immunity would respond to a TC infection with 1.5x10^3^ IFU of *C.t*. serovar D (SvD). For these analyses, we chose only the SC and SIM groups, as the objective was to examine if the resident immunity, observed in the SIM mice, would increase the local post-infection response and the protection. As before, we analyzed the i.v CD45 negative Th1/Th17 T cells in the GT. At day 1 the percentage of CD4 T cells out of all cells in the uGT constituted 7.5% in the SIM group, of which 13% were cytokine positive (**Figures 3A, B**). The percentage of CD4 T cells in the SC group was not different from the non vaccinated group. This showed that no systemic CTH522-specific T cells in the SC had yet been recruited to the GT. To further analyse the local T cells, we plotted the Th1/Th17 cytokine subsets based on their cytokine expression. In particular, this would show the development of effector Th1/Th17 subtypes (22) in SC and SIM animals (indicated by arrows in **Figure 3C**). Effector Th1 T cells were defined as IL-17-, INFγ+, +/- other cytokines, and effector Th17 subtypes as IL-17+, TNFα+, +/- other cytokines. The data demonstrated that at day 1 post infection, the SIM group showed activated multifunctional vaccine-specific CD4 T cells (IL-17+/TNFα+ Th17 or IFNγ+/TNFα+ Th1 T cells) in the GT. SC mice showed some TNFα+ T cells, but these were also seen in non vaccinated animals or vaccinated animals only stimulated with media (**Supplementary Figure 2**).

At day 3 post infection, CD4 T cells in the uGT constituted 10.3% of all cells in the SIM group, compared to 3.9% in the SC group. Cyt+ CD4 T cells constituted 17.6% in the SIM group and 6.1% in the SC group (**Figures 3A, B**). This was in contrast to the spleen and blood where there was no difference between SIM and SC mice (**Supplementary Figure 3**). As observed for day 1 post infection, in the SIM group we observed increased percentage of multifunctional effector CD4 T cells expressing IFNγ/TNFα or IL-17/TNFα (**Figure 3D**). At this time point only a few Th17 T cells co-expressed IFNγ. At day 7 there was a further increase in the percentage of CD4 T cells in the GT in both vaccine groups, as well as in non vaccinated animals. SIM animals showed 21.03% CD4 T cells, whereas SC animals showed 17.8% and non vaccinated 8.7%. Regarding the percentage of Cyt+ CD4 T cells, in SC mice there was a significant increase to 20.5%, whereas the SIM group only showed a minor increase compared to day 3 post infection (**Figures 3A, B**). At this time point the SC and SIM groups showed similar percentages in Cyt+ CD4 T cells. This similarity between SC and SIM animals was also observed in another experiment where we infected the animals at a much later time point (day 154) (**Supplementary Figure 4**), demonstrating efficient recruitment to the GT of systemic CAF01-induced Th1/Th17 T cells, irrespective of their effector-memory/memory status in SC animals post infection. The Th1/Th17 cytokine subsets in the GT were dominated by IFNγ/TNFα or IL-17/TNFα CD4 T cells, and CD4 T cells expressing only IL-17 or IL-17/TNFα (**Figure 3D**).

From day 7 to 14, the SC and SIM groups again showed a different development regarding Cyt+ CD4 T cells. In the SIM group the percentage of Cyt+ CD4 T cells retracted slightly from 20.5% to 17.6%, but in the SC group it increased from 20.5% to 29.3% (**Figure 3B**).

Th17 T cells increased their IFNγ expression in both vaccine groups (**Figures 3E, F**). Thus, while Th1 T cells in the GT increased their IFNγ expression from the onset of the infection, Th17 T cells showed the highest increase in IFNγ expression between day 7 and 14.

Taken together, the SIM group showed a faster local CMI response consisting of multifunctional CD4 T cells. This correlated with an increased activation/recruitment of innate immune cells in the SIM group (dendritic cells, monocytes and neutrophils) (**Supplementary Figure 5**). Finally, the SIM group also exhibited a faster retraction of the local CMI response, compared to SC animals. Between day 3 and 14 the SIM animals showed no increase in % Cyt+ CD4 T cells. In marked contrast, the SC animals showed a 366% increase in the same period. The faster retraction between day 7 and 14 in the SIM group was also reflected in the local population of dendritic cells, monocytes and neutrophils in the SIM group (**Supplementary Figure 5**).

### Protection Against Infection Is Increased in the SIM Group

We next examined if the increased response in SIM animals, compared to SC animals, correlated with increased protection. As a measure of bacterial burden, the bacterial shedding was measured in the uterus at day 7 post a TC infection with 1.5x10^3^ IFU of *C.t* SvD. This time point was chosen based on our previous data (15). The data [depicted as inclusion forming units (IFU)] showed that the SC vaccine induced a significant reduction in the IFU from 4.05 +/- 0.54 (Log_10_ mean +/- SD) to 3.44 +/- 0.40 (corresponding to a reduction of 75% in IFU) (**Figure 5**). However, in the SIM group, the IFUs was further reduced to 2.67 +/- 0.65, corresponding to a further reduction in bacterial numbers of 86% compared to the SC group (or 96% reduction compared to non vaccinated mice). Interestingly, to achieve the increased protection (and a stronger local immunity) compared to the parenteral vaccine, a requirement for the mucosal intrauterine vaccine in the SIM strategy was that it contained the adjuvant CAF01. Thus, supplementing an SC vaccine with a non-adjuvanted uterine administered CTH522 antigen had a negative effect on both the local and systemic immune responses, and compromised the protection against infection (**Supplementary Figure 6**).

### The Local Immune Response in the Genital Tract Following a Second Infection

As repeated infections are often observed in women (5), we next examined whether the double adjuvanted SIM group would also show an advantage in the response against a subsequent infection.

Female B6C3F1 mice were vaccinated three times with two weeks intervals, using the SC or SIM strategy. 14 weeks post immunization the mice received the first TC infection with 1.5x10^3^ IFU of *C.t.* SvD. 14 days post infection the mice received a total of 4 mg azithromycin treatment over a four-day period to clear the infection in all mice. 32 days post infection the mice received a second infection with *C.t.* SvD, and the local immune responses were analyzed at day 3 and 6 post this second infection.

**Figure 4:**
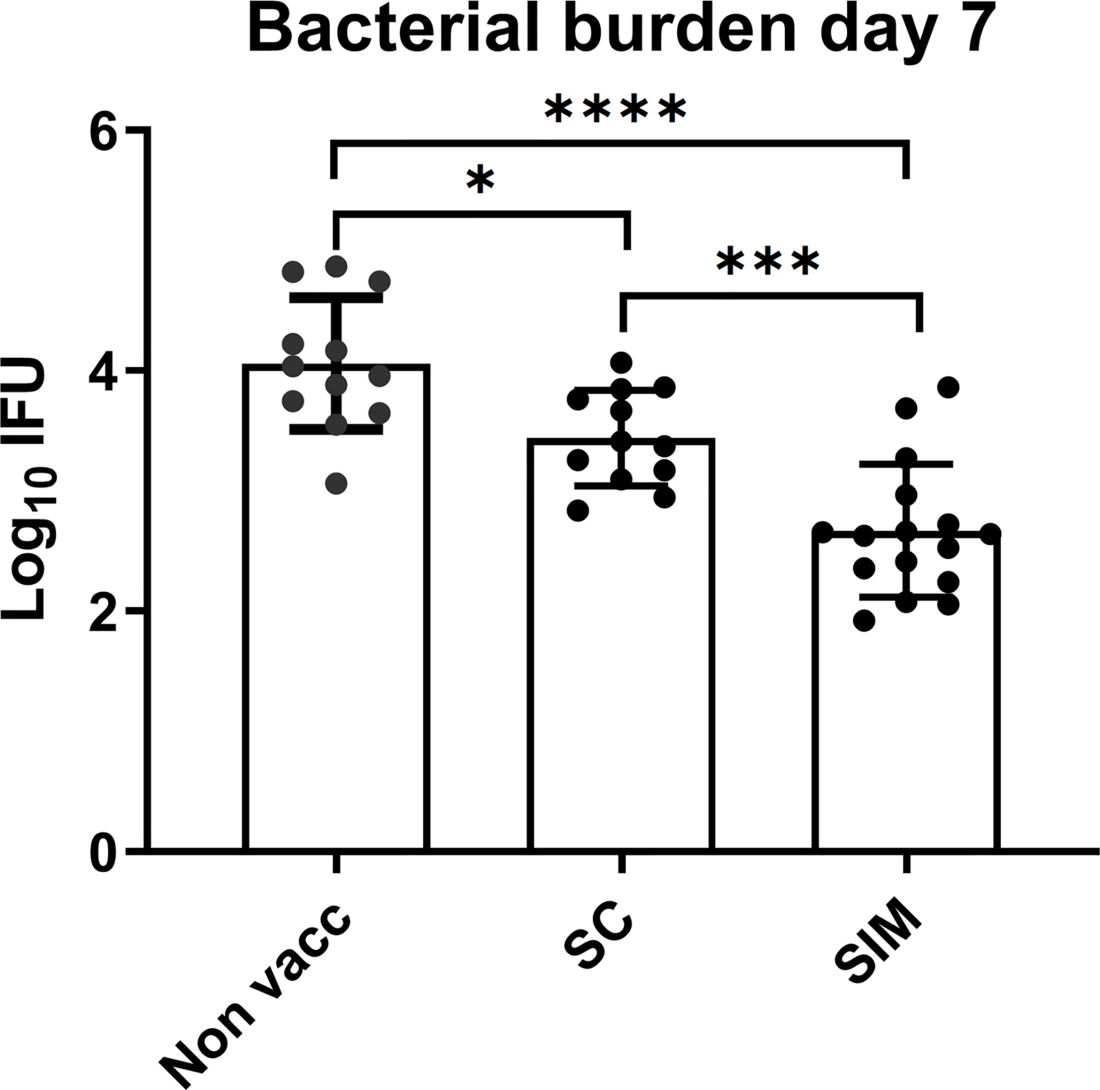
Bacterial levels in the GT in vaccinated or non-vaccinated groups. Groups of female B6C3F1 mice (n=12/16) were vaccinated as indicated, with two weeks intervals. 6 weeks post immunization the mice received a TC infection with 10^3^ IFU of *C.t.* SvD. At day 7 the genital tract bacterial numbers (Log_10_ IFU) were determined. Bars indicate means ± SD. Statistical significance was evaluated by an ANOVA test followed by Tukey’s multiple comparisons using Graphpad Prism 8.3.0. *p < 0.05. ***p < 0.001, ****p < 0.0001. The data shown are representative of 2 experiments.

**Figure 5:**
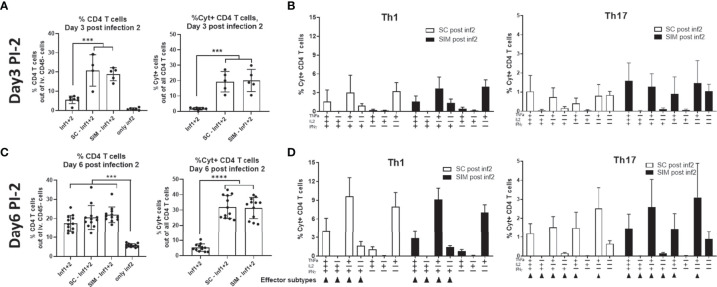
CMI response at day 3 and 6 post a second infection in the genital tract. Groups of female B6C3F1 mice were vaccinated three times as indicated with two weeks intervals. The antigen was CTH522 and the administration route is indicated in the figure. 14 weeks post immunization the mice received the first TC infection (Inf1) with 1.5x10^3^ IFU of *C.t.* SvD. 14 days post infection the mice received 4 mg azithromycin treatment over 4 day-period. 32 days post infection the mice received a second infection (Inf1+2) with 1.5x10^3^ IFU of *C.t.* SvD. **(A, B)** Percentage of CD4+ T cells out of all iv. CD45neg. Cells in the GT and percentage of Cyt+ CD44+ CD4 T cells out of all CD4 T cells in the genital tract at day 3 (n=4) **(A)** or 6 (n=12) **(C)** post infection 2. **(B–D)** Percentages of IL-17 negative CD4 T cells (Th1) and IL-17 positive CD4 cells (Th17) out of all CD4+ T cells in the uGT were analyzed by flow cytometry for the frequency of cytokine subsets (expression of TNFα, IL-2, IFNγ and/or IL-17) in the genital tract at day 3 (n=4) **(B)** or 6 (n=12) **(D)** post infection 2. Small arrows in **(D)** indicate Th1 or Th17 effector subsets. Bars indicate means ± SD. Statistical significance was evaluated by an ANOVA test followed by Tukey’s multiple comparisons. ***p < 0.001, ****p < 0.0001. The data shown are representative of 2 experiments.

In contrast to day 3 post the infection no. 1 (PI-1) which is shown in **Figure 4**, we found that at day 3 post infection no. 2 (PI-2), the percentage of CD4 T cells in the GT was now similar in the SC and SIM groups (20.8% in SC animals and 18.9% in SIM animals) (**Figure 5**). Also in contrast to day 3 PI-1, the percentage of Cyt+ CD4 T cells was also similar in SC and SIM mice (**Figure 5A**). Both SC and SIM mice showed an increase in both CD4 T cells and Cyt+CD44+CD4 T cells compared to mice that did not receive the second infection (**Supplementary Figure 7**). The Th1 and Th17 cytokine subset composition was also similar in SC and SIM animals (**Figure 5B**). The Th1 cytokine subset pattern reminded of the pattern observed at day 7 PI-1, whereas the Th17 cytokine subsets reminded of those observed only at day 14 PI-1 (**Figures 3** and **5**), which suggested a higher activation state of the Th1/Th17 Trm cells after the second infection.

At day 6 PI-2, SC and SIM mice were still similar in terms percentage of CD4 T cells and the percentage of cyt+CD4 T cells, which had now increased to approximately 30% of all CD4 T cells in the GT (**Figure 5C**). In both SC and SIM mice, we observed multifunctional CD4 Th1 and Th17 T cells in the GT and few CD4 Th1/Th17 T cells expressing only IFNγ or IL-17/IFNγ (**Figure 5D**).

A similar CMI response in SC and SIM mice was also observed in the draining iliac lymph node, that showed multi-cytokine expressing Th1 and Th17 T cells (**Supplementary Figure 8**).

**Figure 6:**
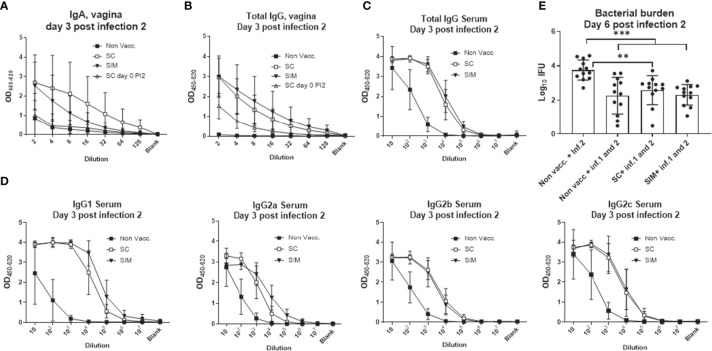
Humoral response and protection following a second infection. Female B6C3F1 mice were vaccinated three times as indicated, with two weeks intervals. 14 weeks post immunization the mice received the first TC infection with 10^3^ IFU of *C.t.* SvD. 14 days post infection the mice received 4 mg azithromycin treatment over 4 days. 32 days post infection the mice received a second infection with 1.5x10^3^ IFU of *C.t.* SvD. **(A, B)** At day 3 post infection 2 (n=4), IgA **(A)** or total IgG **(B)** was measured in the vaginal fluids. As a comparison the IgA levels at day 0 post infection 2 is also shown (inverted solid triangles). **(C, D)** Total IgG **(C)** or IgG subclasses **(D)** at day 3 post infection 2 in serum. **(E)** Genital tract bacterial numbers (Log_10_ IFU) were determined at day 6 post infection 2 (n=12). Bars indicate means ± SD. Statistical significance was evaluated by an ANOVA test followed by Tukey’s multiple comparisons. **p < 0.01, ***p < 0.001. The data shown are representative of 2 experiments.

Concerning the local and systemic humoral response, after the second infection the SC mice showed a boost in the vaginal IgA and IgG response, which was as high as in the SIM group (**Figures 6A, B**). This was in contrast to the IgA levels after the first infection, where the levels were similar to background levels in SC mice (**Supplementary Figure 5B**). In addition, the systemic IgG titer was similar between the SC and SIM groups, as were the IFUs post a second infection (**Figures 6C**, **D**). However, we also noticed that a previous infection also protected strongly against reinfection, demonstrating that in contrast to measuring the immune response against a second infection, the protective effect of a vaccine, given prior to the first infection, could not be measured in this model using bacterial shedding following a second infection as readout (**Figure 6E**).

Taken together, in contrast to the response observed following the first infection, which showed an increased local response in the SIM group, both SC and SIM animals showed a similar fast response after the second infection. This response was dominated by multifunctional Th1 and Th17 effector cells, and a fast increase in the local IgA/IgG response.

## Discussion

It is well known that the genital tract is not an optimal inductive site, and moreover is a site where immune tolerance can develop (8, 23). In agreement with this, although CAF01 is an efficient Th1/Th17 T cell adjuvant, immunization with CTH522/CAF01 in the uterus (IU) did not lead to increased local or systemic cell mediated immunity, and did not increase the systemic IgG titer (**Figures 1**, **2**). Moreover, supplementing a parenteral immunization with unadjuvanted antigen in the uterus had a negative effect on both the local and systemic immunity (**Supplementary Figure 6**). This could be due generation regulatory T cells mediated by the non adjuvanted antigen (8, 24), but this was not shown in this study. In contrast, when combined with a parenteral immunization, an adjuvanted IU immunization did increase both the local genital tract Th1/Th17 response, as well as a local IgA response, prior to infection. Although the SIM animals received a higher total antigen dose (2x5ug) compared to SC animals (5ug), the effect of including the adjuvanted IU antigen was observed in the genital tract and not in the systemic response (**Figures 1**, **2**). Concerning the dose in the SIM group, we have previously tested higher antigen doses of CTH522 and not seen seeding of the GT prior to infection, and compared to SC animals, SIM-PBS animals that also received 10 ug Ag in total, did not show increased T cell numbers in the GT before infection (data not shown), or after infection (**Supplementary Figure 6**). Finally, we have unpublished results where we compared a parenteral vaccine with CAF01/5ug Ag with another group that simultaneously received two parenteral doses of CAF01/5ug Ag, and saw no increased seeding of the GT due to the additional parenteral vaccine. We therefore believe that the effect observed in SIM animals in this study is not merely due to the increase in the total antigen dose. It is not known if the IU antigen is required in the SIM strategy to generate the local CMI response. It could be speculated that the mechanism for Th1/Th17 T cell recruitment is solely due to CAF01-induced expression of cytokines and chemokines from APCs located in the GT (14). Taken together, our results indicated that although an IU immunization could not efficiently prime a response by itself, if adjuvanted it could induce sufficient immunity to alert circulating immunity to be recruited to the genital tract.

Using the SIM strategy to induce GT Th1/Th17 Trm cells, we observed, after infection, a substantial dynamic development in cytokine expression within both the Th1 and Th17 populations, which was driven by the infection. Other studies examining *Chlamydia muridarum* specific Th1 and Th17 T cells, either transgenically generated or induced by vaccine, also showed that Th1/Th17 T cells that express multiple cytokines (IFNγ, IL-2, TNFα and or IL-17) although the local post infection development of these cells was not analysed in these studies (25–27). By comparing the SC and SIM vaccine strategies, we also observed a clear difference in the GT immune response at day 1 and 3 post infection. At day 1 post infection, the SC group was not different from the non vaccinated group, and only the SIM group showed activated multifunctional CD4 T cells in the GT (**Figures 3B, C**). At day 3, the SIM group showed a stronger local T cell response with more developed Th1/Th17 T cell cytokine subsets expressing more effector cytokines. Th17 T cells expressed TNFα and IL-17 and Th1 T cells expressed TNFα and IFNγ (**Figure 3D**). At day 7, the vaccinated SC and SIM groups were not significantly different in terms of Th1/Th17 cytokine subsets. The subsets in the GT were dominated by IFNγ/TNFα or IL-17/TNFα CD4 T cells, and CD4 T cells expressing primarily IL-17 or TNFα (**Figure 3E**). At day 14, although the CD4 T cell levels in the GT were the same in SC and SIM groups, the % Cyt+ CD4 T cells had retracted markedly more in the SIM group compared to SC mice (which instead showed an increase) (**Figures 3A, B**). Thus, the SIM group showed a faster initiation of the local response, and a faster retraction of the response. This applied to both the adaptive and innate immune response. We also noted that at day 14, the Th17 T cells had increased their production of IFNγ to a level where almost 40% of all Th17 T cells co-expressed IFNγ. It is known that fully differentiated Th17 cells can deviate toward a Th1 phenotype (28), acquiring the ability to secrete IFN-γ, which can be useful for host defense against infections, as IFNγ may contribute to a protective response. Th17 T cells have however also been associated with immunopathology. One study showed that Th17 T cells within inflamed joints of rheumatoid arthritis patients had gained expression of IFNγ (and lost expression of IL-17) (29). During a *C.t.* infection, Th17 T cells have also been suggested to be involved in a pathological response (30). However, we recently showed that recruitment to the GT of vaccine-induced Th17 T cells did not correlate with increased pathology, but rather with protection against both infection and chronic pathology (15). It will be important to study if Th17 T cells that start to express IFNγ play a protective, or pathology promoting, role.

Generating tissue-resident memory T cells with the SIM strategy, prior to infection, correlated with an additional reduction of bacterial shedding of 86% compared to a parenteral vaccine. It should be noted that bacterial levels were measured as bacterial shedding, which during an active and ongoing infection most probably is an adequate readout to reflect vaccine-induced protection, even though it may underestimate the total number of bacteria in the tissue. Our result is in agreement with previous studies, in which mucosal immunity, generated by intranasal or intrauterine immunization, was found to increase protection (8, 16, 31, 32). As mentioned above, the increased protection in the SIM group correlated with a faster, and increased, CMI and innate response in the genital tract, as well as with increased IgA levels in the vagina. Given the evidence that mucosal immunity can add to the protection [**Figure 4**, and (8, 16, 31–33)], the question is whether it should be induced by a future *Chlamydia* vaccine. The question is important since a parenteral administration will facilitate delivery of a *C. trachomatis* subunit vaccine, especially when combined with other STI vaccines, and because a parenteral administration is considered more safe and practical than a mucosal vaccine (34). Importantly, several studies have shown sufficient protection with a parenteral immunity (15, 31, 35, 36). Furthermore, the present study showed that although SIM animals had an advantage against the first infection compared to SC animals, no advantage in the immune response could be observed against a second infection (**Figures 5**, **6**). Already at day 3 post a second infection SC animals showed increased numbers of CD4 T cells, and more Cyt+ CD4 T cells in the GT (compared to after the first infection). Both in terms of the percentage of Cyt+ CD4 T cells in the GT, and in the Th1 or Th17 cytokine subset pattern, and regarding the local humoral response, the SC and SIM animals looked similar following a second infection. We conclude that the advantage in the SIM group, regarding a fast vaccine antigen-specific CMI/humoral response, was only apparent after the first infection.

Concerning the animal model used in this study, our results demonstrated that it is useful for testing protection and local immunity after the first infection, as well as vaccine antigen-specific local immunity after the second infection. However, we also found that the first infection induced its own protective response, most probably consisting of infection-induced T cells and antibodies. In addition, activation of innate immunity due the first infection may also have contributed to protection against a subsequent infection. Taken together, it is a not model that is appropriate to measure vaccine-induced protection after the second infection, unless the infection-induced response is decreased, e.g. by applying the second infection later (to reduce trained innate immunity), or by treating the animals with antibiotics after the first infection (to reduce the infection induced adaptive response). We are currently testing several options, with the goal of developing a model to test vaccine-induced protection after repeated infections. A more realistic mucosal vaccine strategy to compare with the parenteral strategy in such a future model could be the intranasal strategy, as this has been shown to induce both systemic and mucosal immunity (8). Finally, a model that might allow us to measure increased protection after a primary and a second infection is the *Chlamydia muridarum* model. In this model it is not required to bypass the cervix to achieve solid infection of uterus and upper genital tract, and both bacterial burden and pathology can be used as readouts when comparing parenteral and mucosal vaccine strategies. Interestingly, in this model a recent study did show that immunity induced by an IN infection did protect against a subsequent vaginal infection, although the data indicated that it was not due to increased seeding of the genital tract caused by the IN vaccine (37).

Taken together, our results suggest that systemic immunity, induced by a parenteral CAF01 adjuvanted vaccine, provide sufficient protection against a first infection with *Chlamydia trachomatis*. Moreover, whereas the SIM strategy induced memory Th1/Th17 T cells in the GT prior to infection, a parenteral vaccine led to long-lived memory Th1/Th17 T cells in the GT after the first infection. This correlated with an increased local response following a second infection to a level seen in mice having received a mucosal SIM vaccine. Given the practical and safety issues associated with mucosal delivery of adjuvanted vaccines, these results are encouraging for future parenteral vaccine strategies against infection with C*hlamydia trachomatis.*

## Data Availability Statement

The original contributions presented in the study are included in the article/**Supplementary Material**. Further inquiries can be directed to the corresponding author.

## Ethics Statement

The experiments were approved by a local animal protection committee at Statens Serum Institut, IACUC, headed by DVM Kristin Engelhart Illigen.

## Author Contributions

Conceived and designed experiments: FF and JD. Performed experiments: NN. Analyzed the data: NN, AO, SG, FF and JD. Drafted and edited the paper: NN, AO, SG, FF, JC, and JD. Each of the listed co-authors made substantial contributions to the work through design and conception, and/or acquisition, analysis, and interpretation of the data. All authors contributed to the article and approved the submitted version.

## Funding

The project was funded by the Danish Research Council, Project ID: DFF – 9039-00191B.

## Conflict of Interest

AWO and FF are co-inventors on a patent application on vaccines against chlamydia [WO2014146663A1]. All rights have been assigned to Statens Serum Institut, a Danish not-for-profit institute under the Ministry of Health.

The remaining authors declare that the research was conducted in the absence of any commercial or financial relationships that could be construed as a potential conflict of interest.

## Publisher’s Note

All claims expressed in this article are solely those of the authors and do not necessarily represent those of their affiliated organizations, or those of the publisher, the editors and the reviewers. Any product that may be evaluated in this article, or claim that may be made by its manufacturer, is not guaranteed or endorsed by the publisher.
