# Supplementary material for: Th1/Th17 T cell Tissue-Resident Immunity Increases Protection, But Is Not Required in a Vaccine Strategy Against Genital Infection With Chlamydia trachomatis
Source: Front Immunol. 2021 Dec 2;12:790463. doi: 10.3389/fimmu.2021.790463 (PMC8674352; doi:10.3389/fimmu.2021.790463)
Supplement: Supplementary file 1 [file DataSheet_1.pdf]

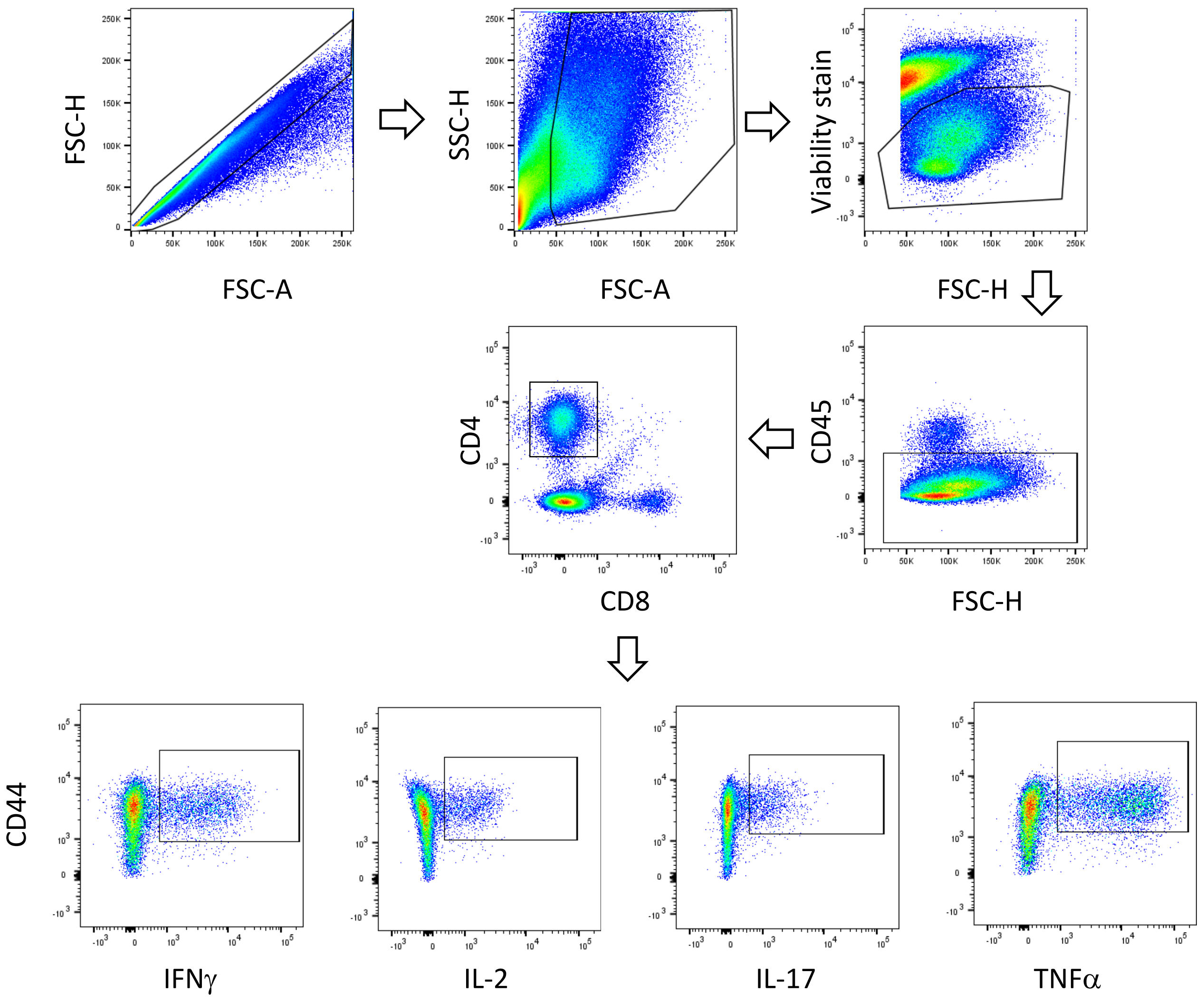


**Supplementary figure 1. Gating strategy for cytokine+ CD4 T cells**

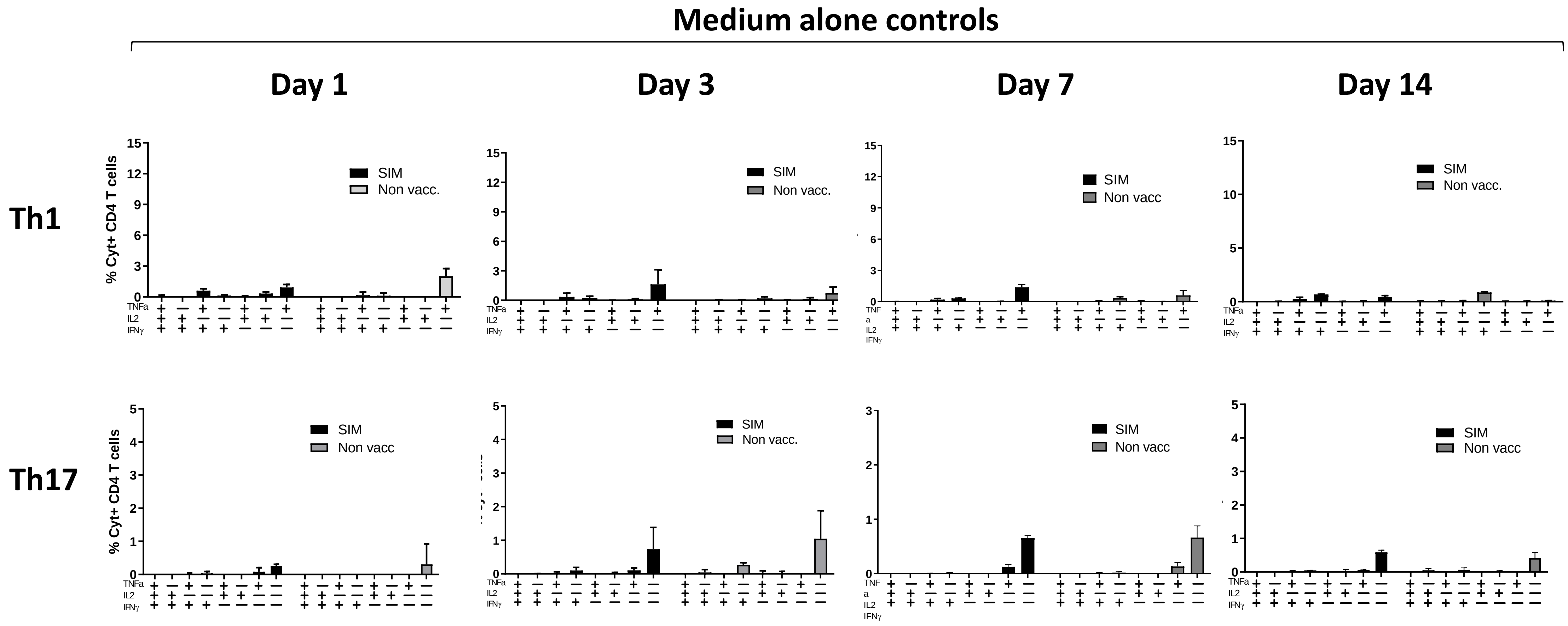


**Supplementary figure 2. Th1 and Th17 cytokine subsets in medium stimulated cells**

Female B6C3F1 mice (n=8, pooled pairwise) were vaccinated three times as indicated with CTH522/CAF01, at two weeks intervals, by the subcutaneous route (SC) or vaccinated by the SC route with CTH522/CAF01 as well as the intrauterine route (with CAF01 adjuvanted CTH522) simultaneously (SIM) or were not vaccinated (Non Vacc). 6 weeks post immunization the mice received a TC infection with $1.5x10^3$ IFU of C.t. SvD. Percentages of IL-17 negative CD4 T cells (Th1) and IL-17 positive CD4 T cells (Th17) out of all CD4+ T cells in the uGT were determined by flow cytometry for the frequency of cytokine subsets (expression of TNFα, IL-2, IFNγ and/or IL-17). Cells cultured in medium and costimulatory antibodies without antigen were used as negative controls.

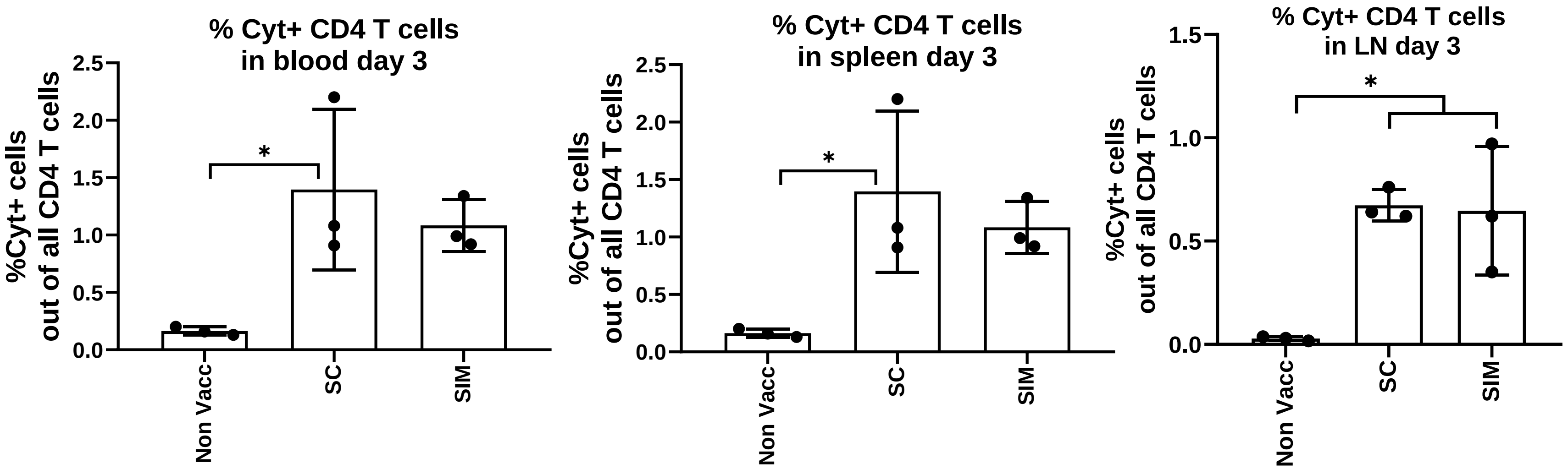


**Supplementary figure 3**. **Percentages of cytokine positive CD4 T cells within CD4 T cell population in blood, spleen or iliac lymphnode**

Female B6C3F1 mice (n=6, pooled pairwise) were vaccinated three times as indicated with CTH522/CAF01, at two weeks intervals, by the subcutaneous route (SC) or vaccinated by the SC route with CTH522/CAF01 as well as the intrauterine route (with CAF01 adjuvanted CTH522) simultaneously (SIM) or were not vaccinated (Non Vacc). 6 weeks post immunization the mice received a TC infection with $10^3$ IFU of *C.t.* SvD

Percentages of cytokine positive (cyt+) CD4 T cells (IFNγ, IL-2, IL-17, and/or TNFα) were determined at day 3 post infection by flow cytometry. Points and error bars indicate means ± SD. Statistical significance was evaluated by an ANOVA test followed by Tukey's multiple comparisons using Graphpad Prism 8.3.0. *$p<0.05$. ***$p<0.001$, ****$p<0.0001$.

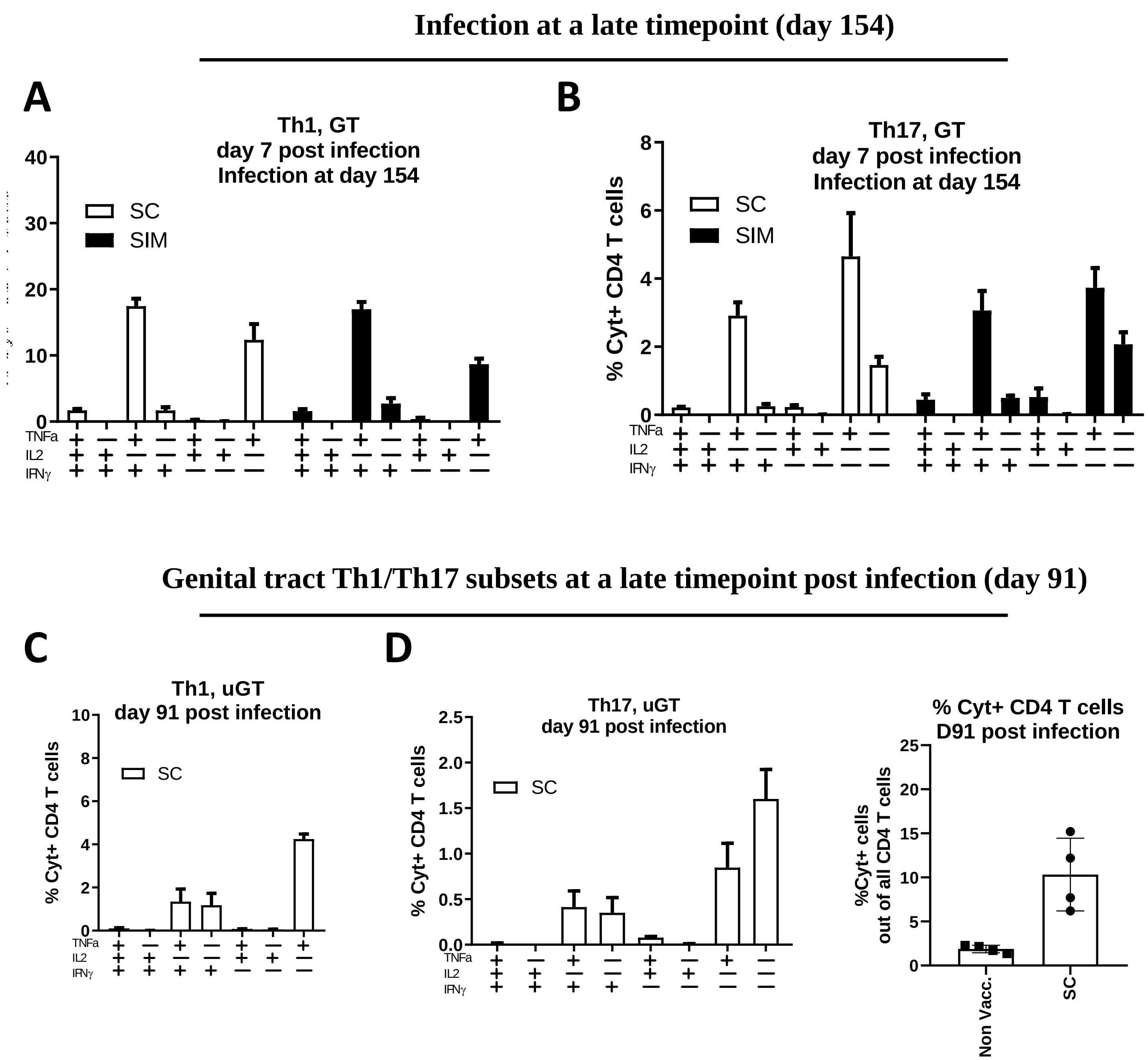


**Supplementary figure 4. Studies of memory response in SC and SIM vaccinated animals.**
**A and B.** Female B6C3F1 mice (n=8) were vaccinated three times as indicated with CTH522/CAF01, at two weeks intervals, by the subcutaneous route (SC) or by the SC and the intrauterine route simultaneously (SIM). At day 154 post immunization the mice received the first TC infection with $10^3$ IFU of *C.t*. SvD. Percentage of Th1 and Th17 cytokine subsets out of all CD4 T cells in the GT is shown**. C and D**. Mice were vaccinated as above. 3 weeks post immunization the mice received a TC infection with $10^3$ IFU of *C.t.* SvD. and percentage of Th1 and Th17 cytokine subsets out of all CD4 T cells in the GT (C) or cytokine positive CD4 T cells were analyzed.

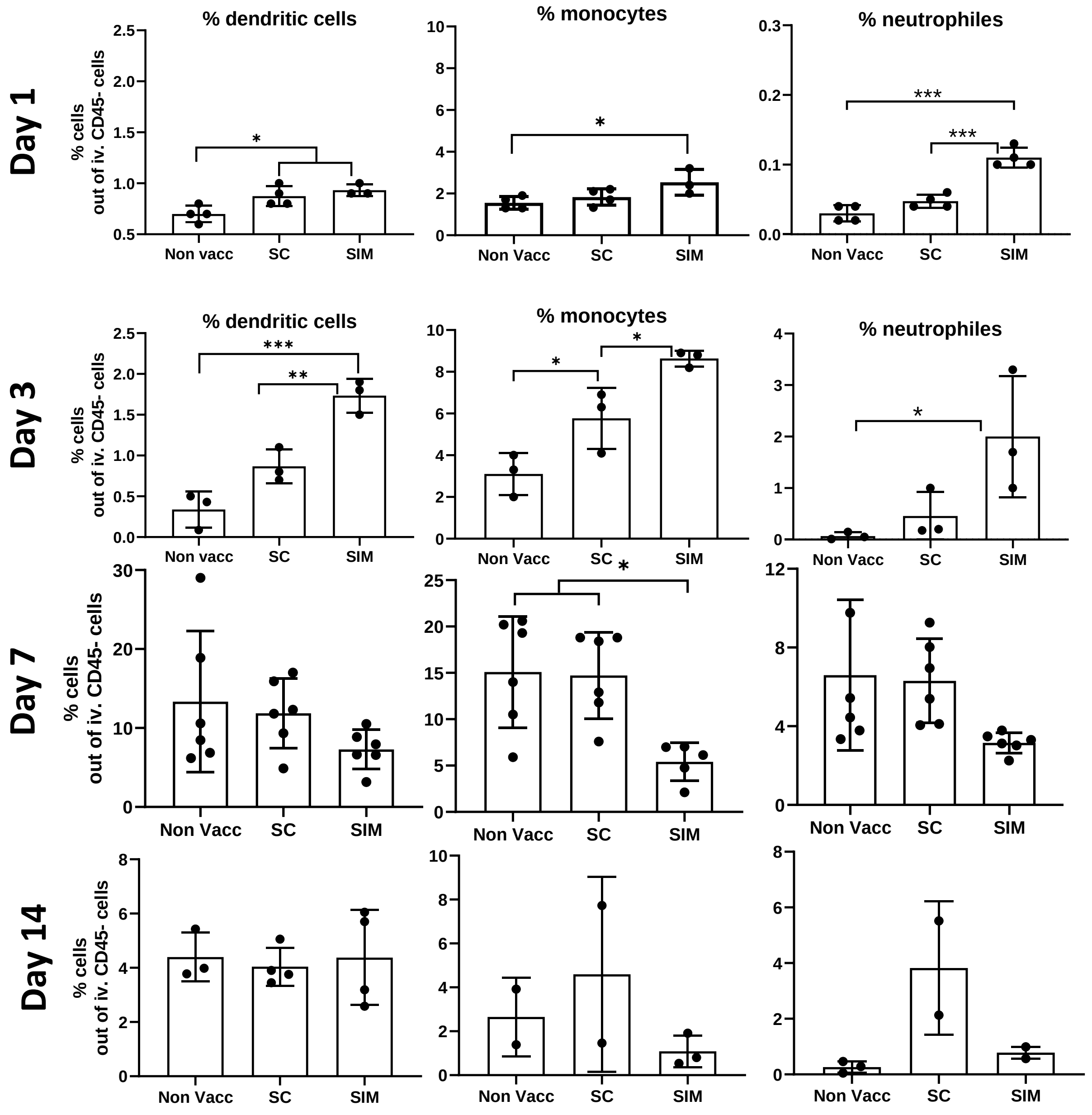


**Supplementary figure 5. The innate response at day 1-14 post infection**

Female B6C3F1 mice (n=6/8 pooled pairwise) were vaccinated three times as indicated with CTH522/CAF01, at two weeks intervals, by the subcutaneous route (SC) or vaccinated by the SC route with CTH522/CAF01 as well as the intrauterine route (with CAF01 adjuvanted CTH522) simultaneously (SIM) or were not vaccinated (Non Vacc). 6 weeks post immunization the mice received a TC infection with $1.5x10^3$ IFU of *C.t.* SvD and the percentage of the indicated immune cells (CD11b+,CD11c+ dendritic cells, CD11b+,CD11c-,Ly6G- monocytes or CD11b+, Ly6G+ neutrophiles) were determined in the GT at the indicated timepoints.
Points and error bars indicate means ± SD. Statistical significance was evaluated by an ANOVA test followed by Tukey's multiple comparisons using Graphpad Prism 8.3.0. *p<0.05, **p<0.01, ***p<0.001.

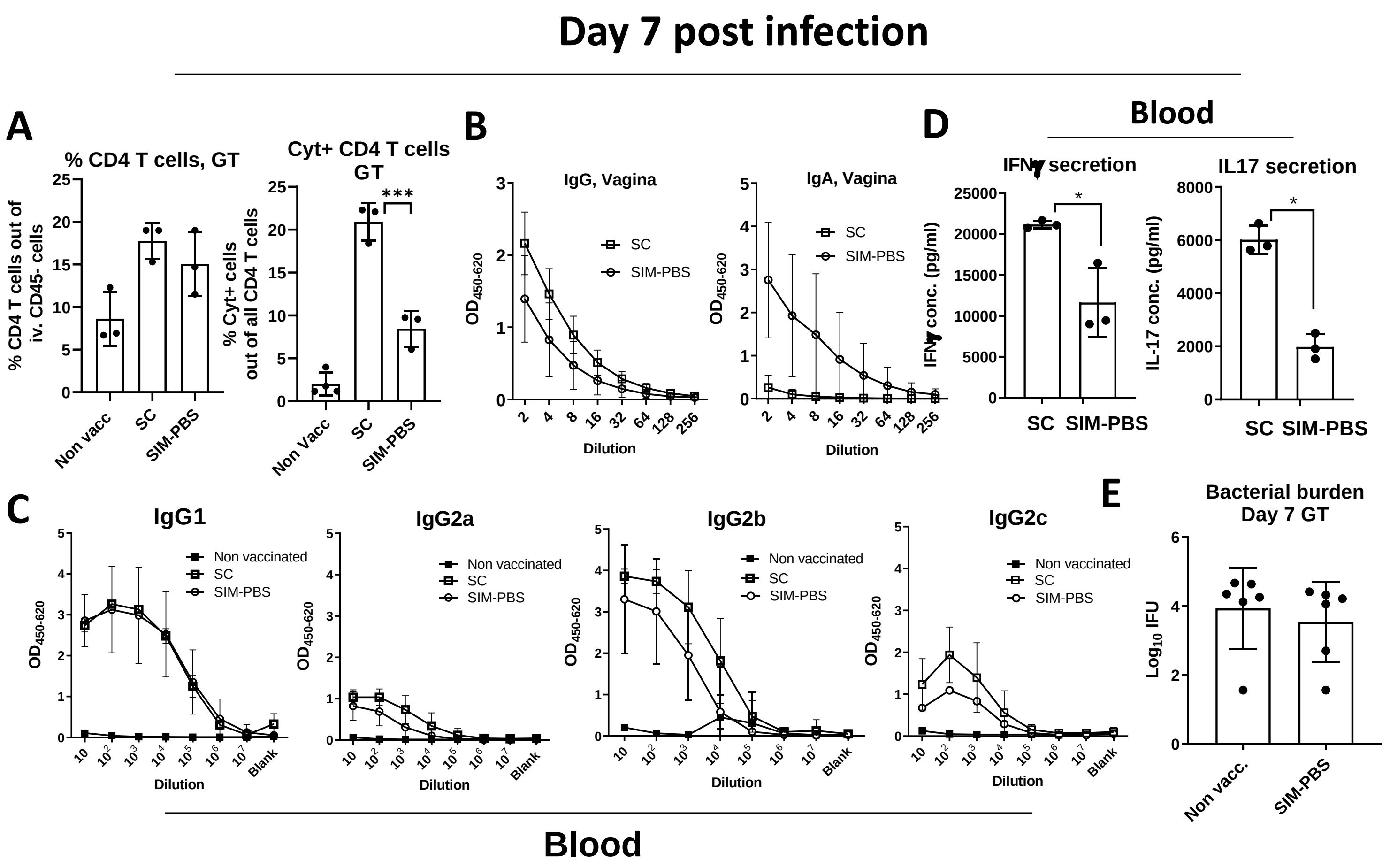


**Supplementary figure 6. Testing the SIM-PBS vaccine strategy**

**A.** Female B6C3F1 mice (n=8) were vaccinated three times as indicated with CTH522/CAF01, at two weeks intervals, by the subcutaneous route (SC) or by the SC with CTH522/CAF01 and the intrauterine route (with non adjuvanted CTH522) simultaneously (SIM), or were not vaccinated (Non Vacc). 6 weeks post immunization the mice received a TC infection with $1.5 \times 10^3$ IFU of *C.t.* SvD. The percentages of CD4 T cells, and cytokine positive (cyt+) CD4 T cells (IFNγ, IL-2, IL-17, and/or TNFα) in uGT was determined at day 7 post infection by flow cytometry. **B**. Total CTH522-specific IgG and IgA levels in the vagina at day 7 post infection. **C.** CTH522-specific IgG1/IgG2a/IgG2b/IgG2c levels were determined in the serum. **D.** Blood PBMCs were stimulated with CTH522 for 72 hours and secreted IFNγ or IL-17 was measured by ELISA. **E.** Groups of female B6C3F1 mice (n=6) were vaccinated as indicated, with two weeks intervals. 6 weeks post immunization the mice received a TC infection with $10^3$ IFU of *C.t.* SvD**.** At day 7 the genital tract bacterial numbers ($Log_{10}$ IFU) was determined. SIM-PBS imply that the mice received CTH522/CAF01 subcutaneously together with non-adjuvanted CTH522 given intrauterine.
Bars indicate means ± SD. Statistical significance was evaluated by an unpaired *t*-test or by ANOVA test followed by Tukey's multiple comparisons using Graphpad Prism 8.3.0. $*p<0.05$. $***p<0.001$, $****p<0.0001$.

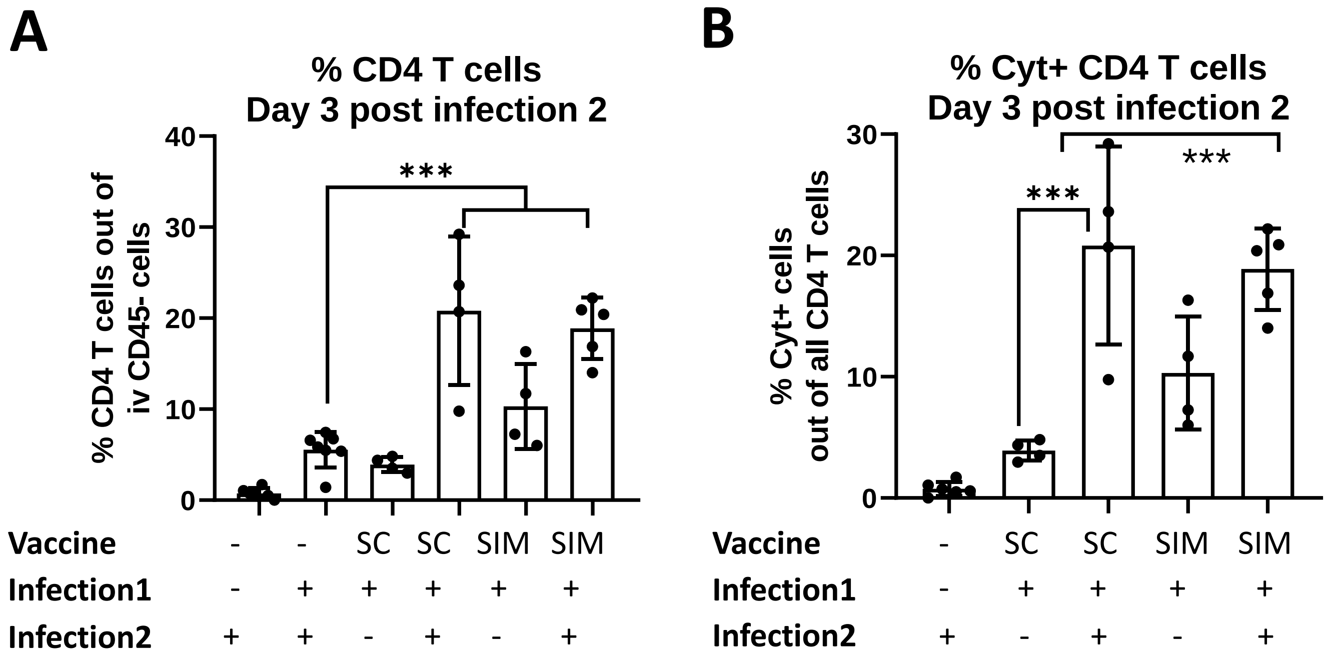


**Supplementary figure 7. SC and SIM strategy at day 3 post a second infection**
Female B6C3F1 mice (n=8) were vaccinated three times as indicated with CTH522/CAF01, at two weeks intervals, by the subcutaneous route (SC) or by the SC and the intrauterine route simultaneously (SIM). 14 weeks post immunization the mice received the first TC infection with $1.5x10^3$ IFU of C.t. SvD. 14 days post infection the mice received 4 mg azithromycin treatment over 4 days. 32 days post infection the mice received a second infection with $1.5x10^3$ IFU of C.t. SvD. **A and B.** Percentage of CD4+ T cells out of all iv. CD45neg cells in the genital tract **(A)** and frequency of cyt+ CD44+ CD4 T cells out of all CD4 T cells in the genital tract **(B)**. Points and bars indicate means ± SD. Statistical significance was evaluated by an ANOVA test followed by Tukey's multiple comparisons using Graphpad Prism 8.3.0. *p<0.05.

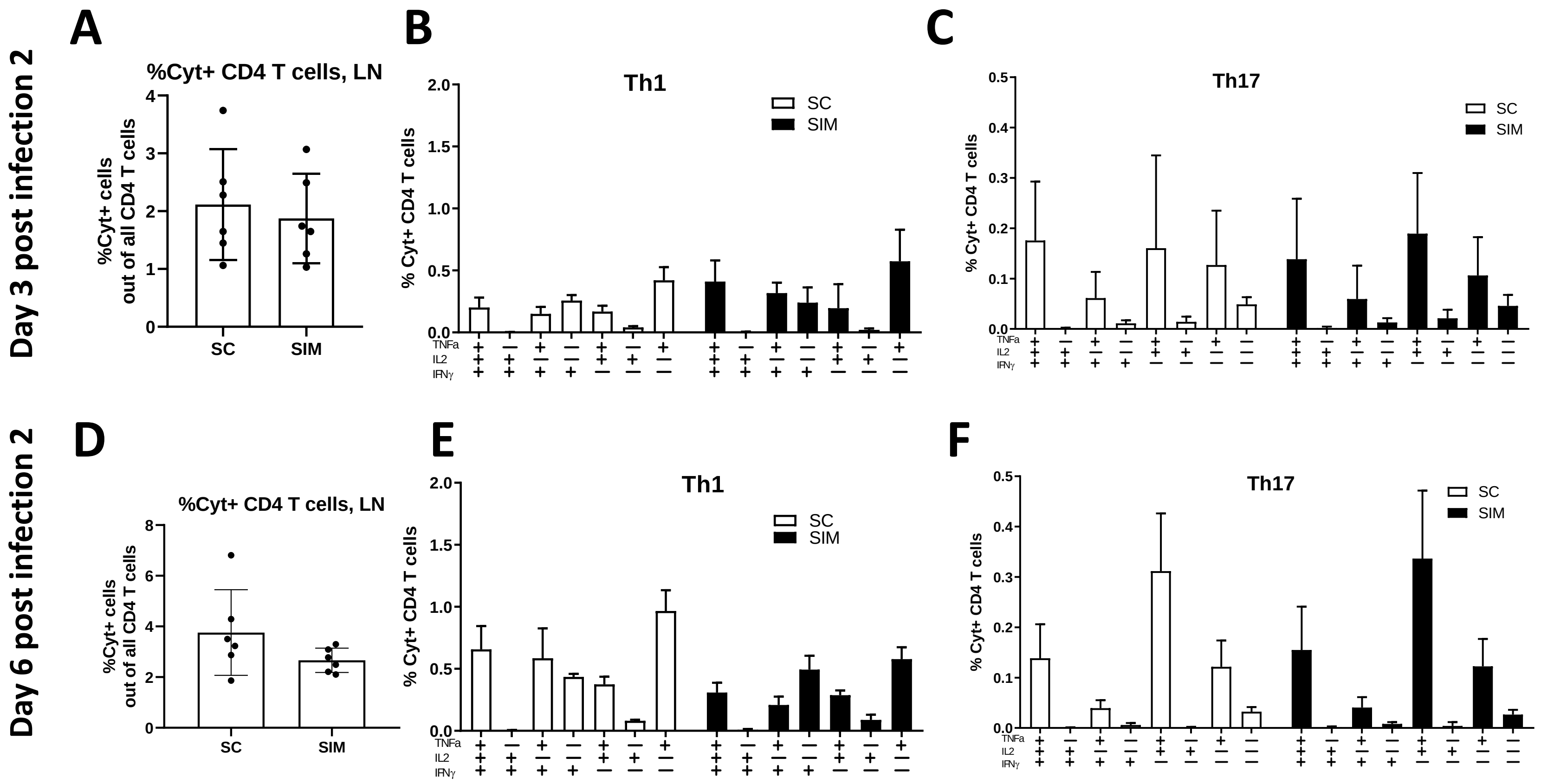


**Supplementary figure 8. SC and SIM strategy at day 3 and 6 post a second infection in the Iliac Lymph node**
Female B6C3F1 mice (n=8) were vaccinated three times as indicated with CTH522/CAF01, at two weeks intervals, by the subcutaneous route (SC) or by the SC and the intrauterine route simultaneously (SIM). 14 weeks post immunization the mice received the first TC infection with 1.5x103 IFU of *C.t*. SvD. 14 days post infection the mice received 4 mg azithromycin treatment over 4 days. 32 days post infection the mice received a second infection with 1.5x103 IFU of C.t. SvD. **A-C.** day 3 post infection 2 (n=6). **D-F** day 6 post infection 2 (n=12, pooled pairwise). **A and D.** Percentage of CD4+ T cells out of cells in iliac lymphnode (LN) and frequency of cyt+ CD44+ CD4 T cells in LNs. **B, C, D, F** show percentage of Th1 and Th17 cytokine subsets out of all CD4 T cells in the iliac lymphnode.
